# Comparative Effectiveness and Safety of Hem-o-Lok® Clips Versus Endoloops for Appendiceal Stump Closure in Laparoscopic Appendectomy: A Randomized Controlled Trial in a Tertiary Care Center in Eastern Uttar Pradesh, India

**DOI:** 10.7759/cureus.114117

**Published:** 2026-08-07

**Authors:** Mukul Singh, Darshan Jalwal, Gaurav Gupta, Harikesh Yadav, Ravi Gupta, Dharmendra K Pipal, Raja Murtaza, Jagani Harsha Sai, Tanushree Trivedi, Rajesh Kannan

**Affiliations:** 1 General Surgery, All India Institute of Medical Sciences, Gorakhpur, IND

**Keywords:** appendiceal stump closure, appendicitis, endoloop, hem-o-lok clip, laparoscopic appendectomy, randomized controlled trial

## Abstract

Background: Secure closure of the appendiceal stump is a key determinant of safe laparoscopic appendectomy. Endoloop (Ethicon, Johnson & Johnson, Cincinnati, OH) ligatures and Hem-o-Lok® (Teleflex Medical, Wayne, PA) clips are both used for this purpose, but current guidelines do not favor one over the other technique.

Objective: To compare Hem-o-Lok® clips and endoloops for appendiceal stump closure with respect to operative time, intraoperative complications, postoperative complications, and hospital stay.

Methodology: This prospective randomized controlled trial was conducted at AIIMS, Gorakhpur, India. Sixty-eight patients undergoing laparoscopic appendectomy for uncomplicated appendicitis were randomly allocated into either the Hem-o-Lok® clip group (*n* = 34, 50%) or the endoloop group (*n* = 34, 50%). All patients diagnosed with appendicitis who fulfilled the study's inclusion criteria were sent back to the registration counter, where the counter-in-charge assigned them to the study groups using sealed, concealed envelopes. Sample size was calculated with G*Power (*d* = 0.7, α = 0.05, power = 0.80, Heinrich-Heine-Universität Düsseldorf, Düsseldorf, Germany). Groups were compared using the independent-samples t-test/Mann-Whitney U test (continuous variables) and the chi-square test (categorical variables); *P* < 0.05 was considered significant.

Results: Groups were well matched for age, sex, body mass index (BMI), comorbidity, prior abdominal surgery, and appendix diameter (all *P* > 0.05). Mean operative time was shorter with Hem-o-Lok® clips (45.94 ± 9.87 vs. 59.24 ± 11.66 minutes; *t* = −5.08, *P* < 0.001), an advantage seen in both emergency (*t* = −3.28, *P* = 0.005) and elective cases (*t* = −4.04, *P* < 0.001). Hospital stay was modestly shorter with clips (4.85 ± 0.89 vs. 5.44 ± 1.11 days; *t* = −2.42, *P* = 0.019). Minimal intraoperative bleeding, drain placement, or conversion occurred in either group, and no complications occurred in either arm over 40 days of follow-up.

Conclusions: Hem-o-Lok® clips reduce operative time and modest hospital stay compared with endoloops in uncomplicated laparoscopic appendectomy. No increase in observed early complications was identified, although the study was not powered to assess uncommon safety outcomes, and they are a comparatively safe, efficient alternative.

## Introduction

Acute appendicitis is among the commonest surgical emergencies worldwide, with a lifetime risk of roughly 7%-8% [1,2]. Since Charles McBurney first described an operative approach in 1889 [1], management has evolved substantially, and laparoscopic appendectomy is now the preferred approach in most settings, offering less pain, fewer wound infections, and faster recovery than open surgery [2,3]. Unlike open appendectomy, the laparoscopic approach requires a dedicated method of securing the appendiceal stump. Inadequate closure risks leakage of contaminated luminal content, with resulting abscess, peritonitis, or sepsis; intra-abdominal abscess is in fact more common after laparoscopic than open appendectomy (approximately 2%-4% vs. 0.5%-1%), a difference partly attributed to variability in stump closure technique [3]. Endoloop (Ethicon, Johnson & Johnson, Cincinnati, OH) ligatures are inexpensive and versatile but demand intracorporeal knot-tying skill and have been linked to slippage and infective complications [4]; linear staplers seal and divide the base reliably but need a larger port and add cost without a clear benefit in uncomplicated disease [5].

Non-absorbable polymeric locking clips, of which the Hem-o-Lok® (Teleflex Medical, Wayne, PA) clip is the most widely used, were adapted from vascular surgery for appendiceal stump closure in the mid-2000s [6]. Their self-locking mechanism resists slippage, application is rapid, and they are deliverable through a standard port for bases up to about 16 mm [7,8].

Despite this, the 2020 guidelines of the World Society of Emergency Surgery (WSES) regard endoloops and polymeric clips as comparably effective and state that clips, endoloops, and staplers are all acceptable depending on appendix base diameter, inflammation, surgeon experience, and availability, but stop short of recommending either technique.

Although several randomized studies and recent meta-analyses have demonstrated comparable safety between polymeric clips and endoloops, additional prospective evidence from diverse healthcare settings remains valuable because operative practices, resource availability, and patient characteristics may differ.

We therefore conducted a randomized controlled trial comparing non-absorbable polymeric locking ligation system clips with endoloops for appendiceal stump closure, with the primary objective of comparing operative time, and the secondary objective of comparing intraoperative and postoperative complications, hospital stay, and early recovery.

## Materials and methods

Study design and setting

**Figure 1:**
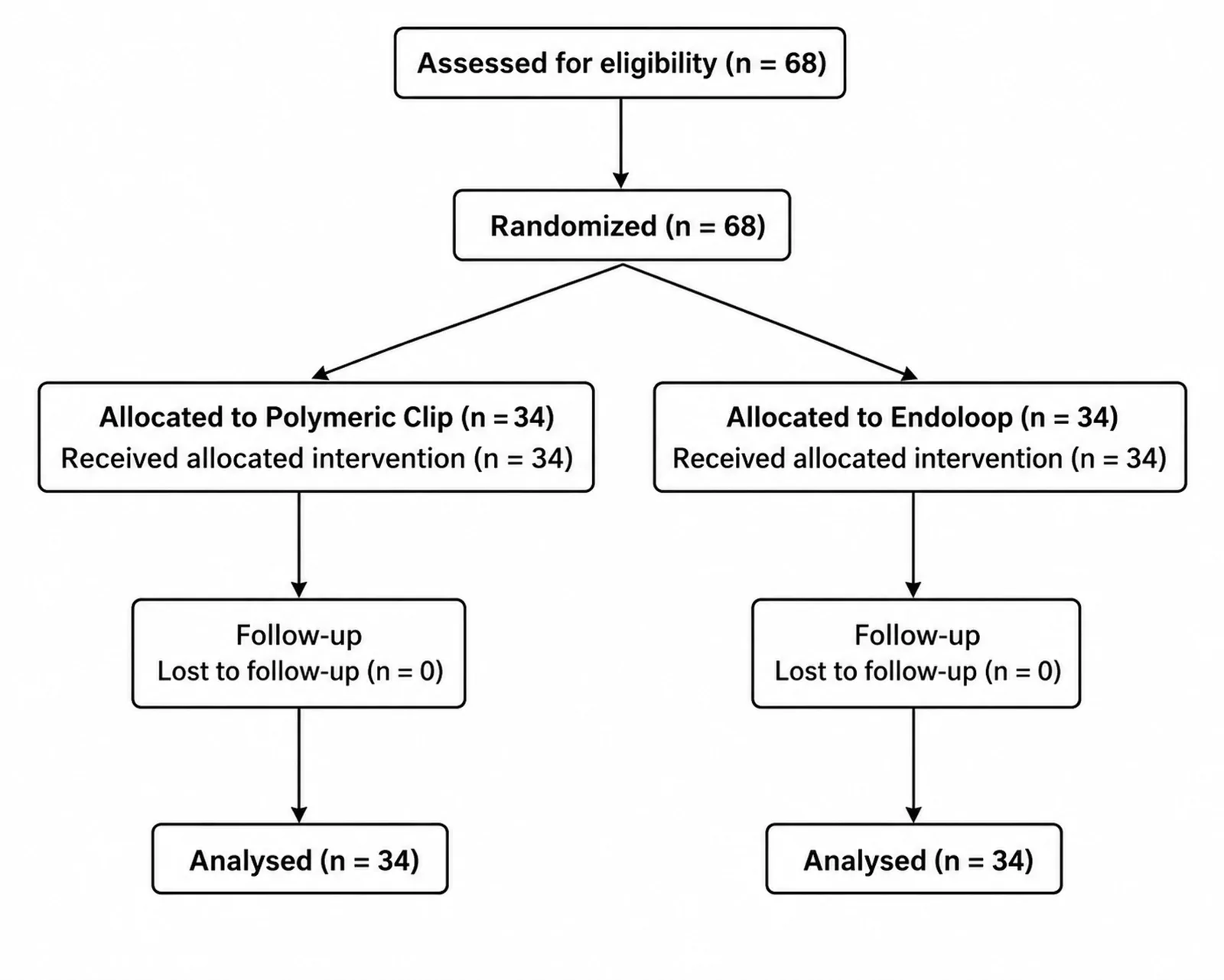
CONSORT-type flow diagram. No randomized patients were excluded, and all participants completed the study and were included in the final analysis. CONSORT, Consolidated Standards of Reporting Trials

This prospective, randomized, parallel-group trial was conducted in the Department of General Surgery after approval from the Institutional Ethics Committee (Institutional Human Ethics Committee, All India Institute of Medical Sciences, Gorakhpur, India, 273008; Approval No. IHEC/AIIMS-GKP/BMR/307/2024; approval date: 26/09/2024) and registration with the Clinical Trials Registry-India (CTRI) (Registration No. CTRI/2024/10/075568; registered on October 21, 2024). Sample collection was started in November 2024. Written informed consent was obtained from all participants. Participant flow is summarized in Figure 1 (Consolidated Standards of Reporting Trials (CONSORT)). The study was concluded in April 2026.

Study population

Patients scheduled for laparoscopic appendectomy for uncomplicated appendicitis, who provided informed consent, were included. Patients with perforated or necrotizing appendicitis, pregnancy, conversion to open surgery, a concomitant procedure, or refusal to participate were excluded.

Sample size

Sample size was calculated in G*Power (Heinrich-Heine-Universität Düsseldorf, Düsseldorf, Germany) for an independent-samples t-test, using an anticipated effect size (*d*) of 0.7, α = 0.05, and power = 0.80 (1:1 allocation), giving 34 patients per group (*N* = 68; achieved power 0.812).

The anticipated effect size (*d* = 0.7) was chosen a priori as a conservative estimate of a medium-to-large between-group difference, informed by the consistent direction of effect reported across the comparative literature on clip versus endoloop appendiceal stump closure, in which Hem-o-Lok® clips have repeatedly been associated with shorter operative time and hospital stay than endoloops [10,11]. This assumption is further corroborated by a subsequently published comparative study, which reported operative time (61.46 ± 10.76 vs. 68.03 ± 8.80 minutes) and hospital stay (3.63 ± 0.77 vs. 4.20 ± 0.93 days) for polymer clips versus endoloop ligatures, corresponding to a calculated Cohen's *d* of approximately 0.67-0.68 for both outcomes - closely matching our a priori assumption [12].

Randomization and blinding

Patients were randomly allocated into either the Hem-o-Lok® clips group (*n* = 34, 50%) or the endoloop group (*n* = 34, 50%). All patients diagnosed with appendicitis who fulfilled the study's inclusion criteria were sent back to the registration counter, where the counter-in-charge assigned them to the study groups using sealed, concealed envelopes. Neither the surgeon nor the patient could be blinded to the technique used; however, the postoperative assessor, statistician, and outcome assessor were blinded.

Study variables and follow-up

Baseline variables included age, sex, BMI, comorbidities, and surgical/hospitalization history. Intraoperative variables included case urgency, appendix diameter, bleeding, drain use, operative time (defined as the interval from placement of the first laparoscopic port to completion of skin closure and recorded by the floor nurse), and conversion.

Postoperative variables included time to oral intake, flatus, discharge, and suture removal, as well as complications (stump leak, surgical site infection, collection, and obstruction). Follow-up continued until postoperative day 40.

Surgical Technique

**Figure 2:**
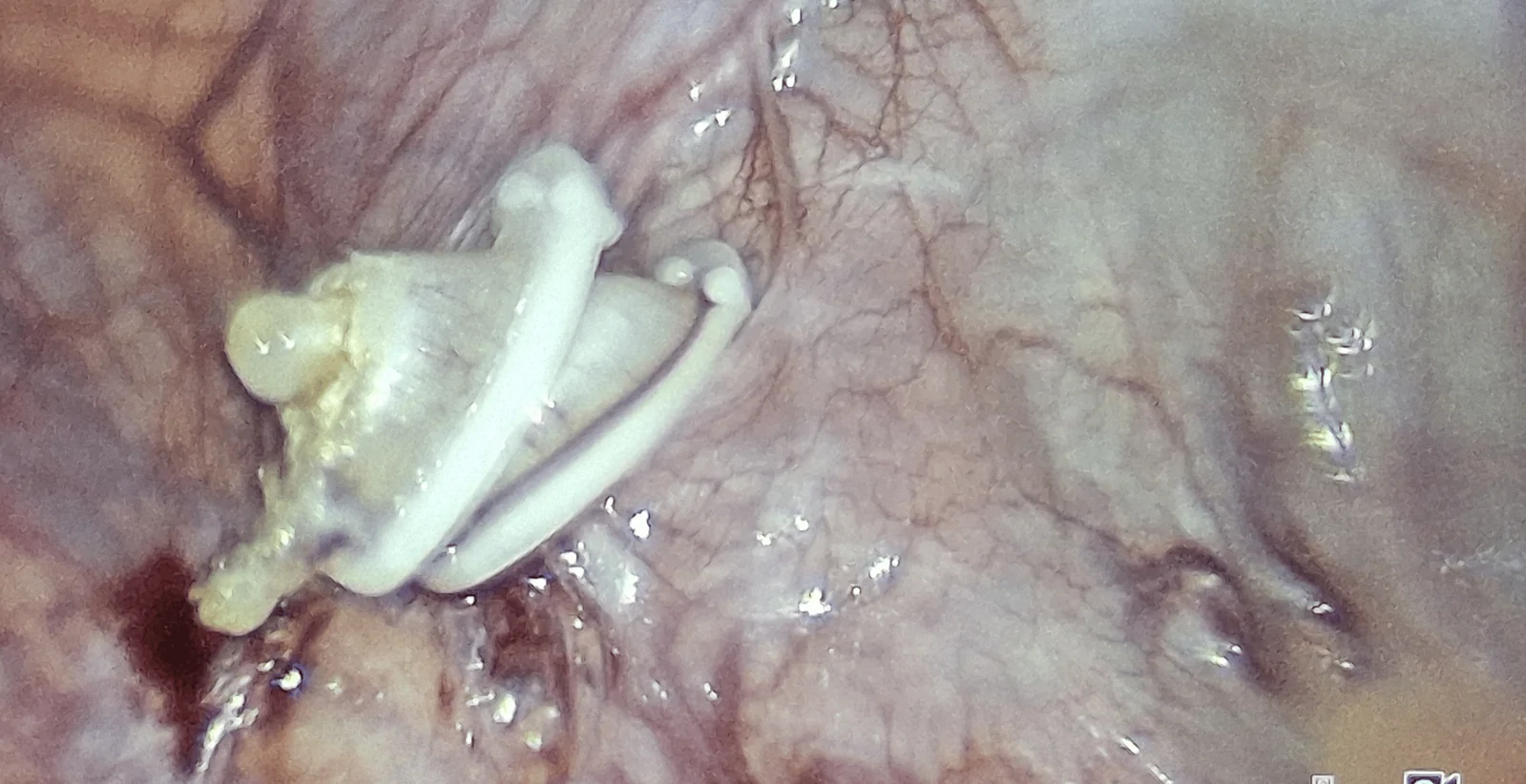
Hem-o-Lok® clips. Representative image of the Hem-o-Lok® clips used for appendiceal stump closure during laparoscopic appendectomy.

**Figure 3:**
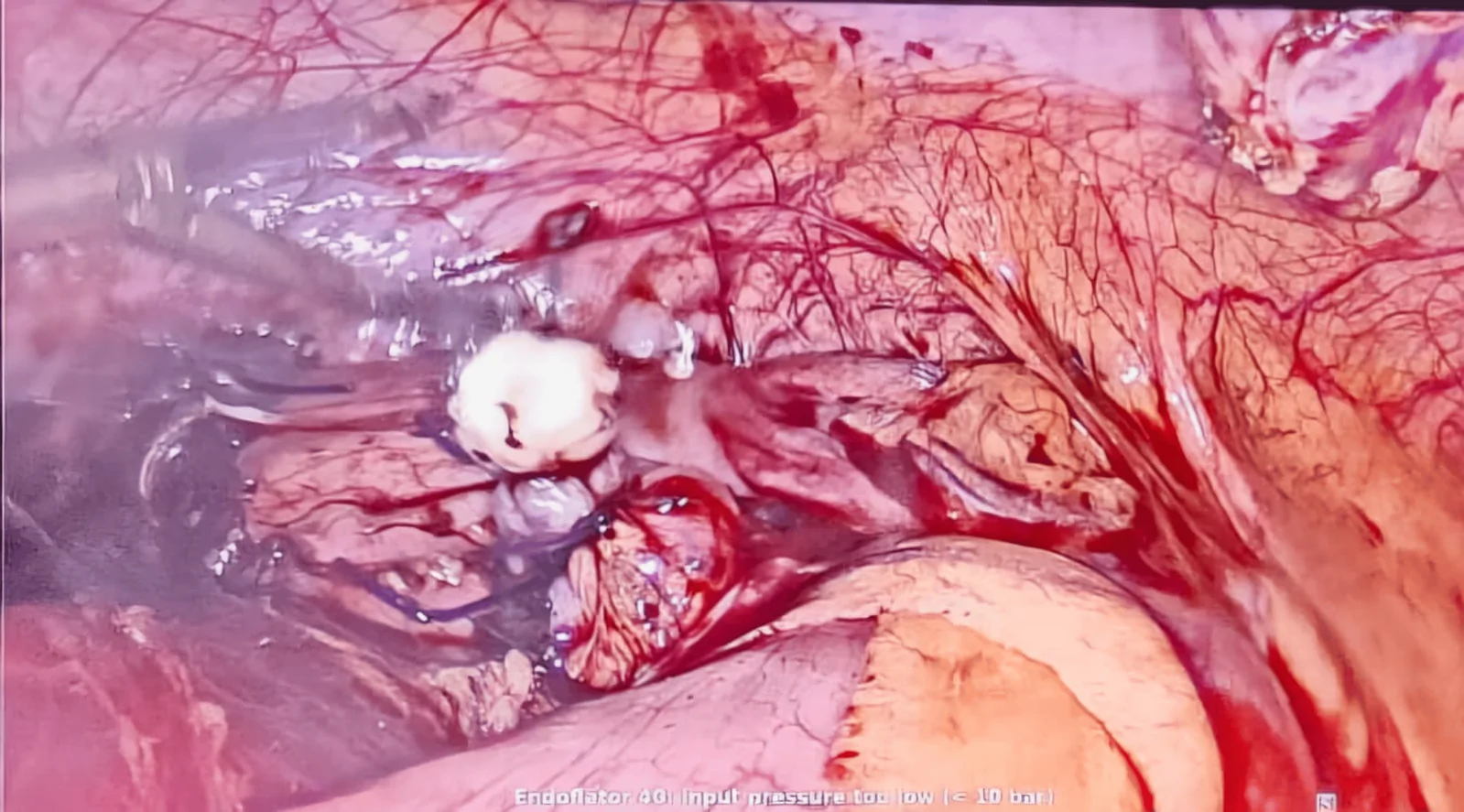
Endoloop ligatures. Representative image of the Endoloop ligatures used for appendiceal stump closure during laparoscopic appendectomy.

All surgeries were performed by a total of four surgeons with similar laparoscopic experience (>5 years), and both groups were equally distributed among the surgeons. All procedures were performed under general anesthesia with endotracheal intubation. Pneumoperitoneum was created after induction of anesthesia via the open technique using a 10-mm umbilical port. The umbilical port was then used to insert a 10-mm 30° laparoscope, and under direct vision, a 5-mm suprapubic port and a 10-mm port were inserted in the left lower quadrant. The laparoscope was then transposed to the left lower quadrant port, and the two remaining ports were used as working ports, with the umbilical port serving as the primary (service) port. Patient positioning was 15-20° Trendelenburg with a 10°-15° left lateral tilt. The appendix was grasped and retracted, and dissection of the mesoappendix was performed using harmonic cautery. In the Hem-o-Lok® clips (Figure 2) group, two clips were placed on the stump side and one on the appendix side, and the appendix was divided using harmonic cautery. In the Endoloop group, a 2-0-sized knot was tied, with two knots placed on the stump side and one on the appendix side (Figure 3); the appendix was then divided and removed using an endobag. For both groups, the 10-mm trocar site was closed with Vicryl sutures at the fascial level, and a skin stapler was used for skin closure.

Statistical analysis

Continuous variables (mean ± SD) were compared using the independent-samples t-test (or Mann-Whitney U test where indicated); categorical variables were compared using the chi-square test. *P* < 0.05 was considered statistically significant throughout.

Normality of continuous variables was assessed using the Shapiro-Wilk test together with visual inspection of histograms and Q-Q plots; the independent-samples t-test (with Welch's correction where variances were unequal on Levene's test) was used for normally distributed variables, and the Mann-Whitney U test was used where this assumption was not met. There were no missing data for any baseline, intraoperative, or postoperative variables: all 68 enrolled patients had complete data recorded at every follow-up point (discharge, suture removal, and postoperative day 40), so no imputation was required. All statistical analyses were performed using SPSS version 27.0 (IBM Corp., Armonk, NY)

## Results

Sixty-eight patients were randomized 1:1 (34 Hem-o-Lok® clips, 34 endoloops; Figure 1). All completed the allocated procedure and 40-day follow-up, with no loss to follow-up or crossover.

The groups were statistically comparable at baseline (Table 1): mean age, height, BMI, sex distribution, appendix diameter, comorbidity, and prior abdominal surgery did not differ significantly (all P > 0.05). The proportion of elective versus emergency procedures was also balanced (Hem-o-Lok® clips: 79.4% elective vs. endoloop: 70.6% elective). A history of previous hospitalization was considered a surrogate marker for prior conservative management of an appendicular lump, which may increase the likelihood of intra-abdominal adhesions and potentially make the operative procedure more challenging. However, this variable was equally distributed between the two study groups, indicating no significant baseline difference that could have influenced the surgical outcomes.

**Table 1:** Baseline demographic, anthropometric, and clinical characteristics. Continuous variables were compared using the independent-samples (Welch) t-test; categorical variables were compared using the chi-square (χ²) test with 1 degree of freedom. Degrees of freedom (df) ranged from 63 to 66 for Welch-adjusted t-tests.

Parameter	Polymeric clip (*n *= 34)	Endoloop (*n *= 34)	Test statistic	*P*-value
Age, years (mean ± SD)	25.76 ± 11.19	28.26 ± 13.66	*t* = -0.83	0.412
Height, cm (mean ± SD)	160.97 ± 12.58	157.09 ± 13.38	*t* = 1.23	0.222
BMI, kg/m² (mean ± SD)	23.15 ± 3.32	23.38 ± 3.34	*t* = −0.28	0.772
Male sex, *n* (%)	18 (52.9)	15 (44.1)	χ² = 0.53	0.467
Female sex, *n* (%)	16 (47.1)	19 (55.9)	χ² = 0.53	0.467
Elective procedure, *n* (%)	27 (79.4)	24 (70.6)	χ² = 0.71	0.401
Appendix diameter, mm (mean ± SD)	8.06 ± 2.59	8.35 ± 2.45	*t* = −0.47	0.557
Any comorbidity, *n* (%)	4 (11.8)	6 (17.6)	χ² = 0.47	0.493
Previous abdominal surgery, *n* (%)	5 (14.7)	6 (17.6)	χ² = 0.11	0.742
Previous hospitalization, *n* (%)	13 (38.2)	14 (41.2)	χ² = 0.06	0.804

No patient in either group had significant intraoperative bleeding, required a drain, or was converted to open surgery.

**Figure 4:**
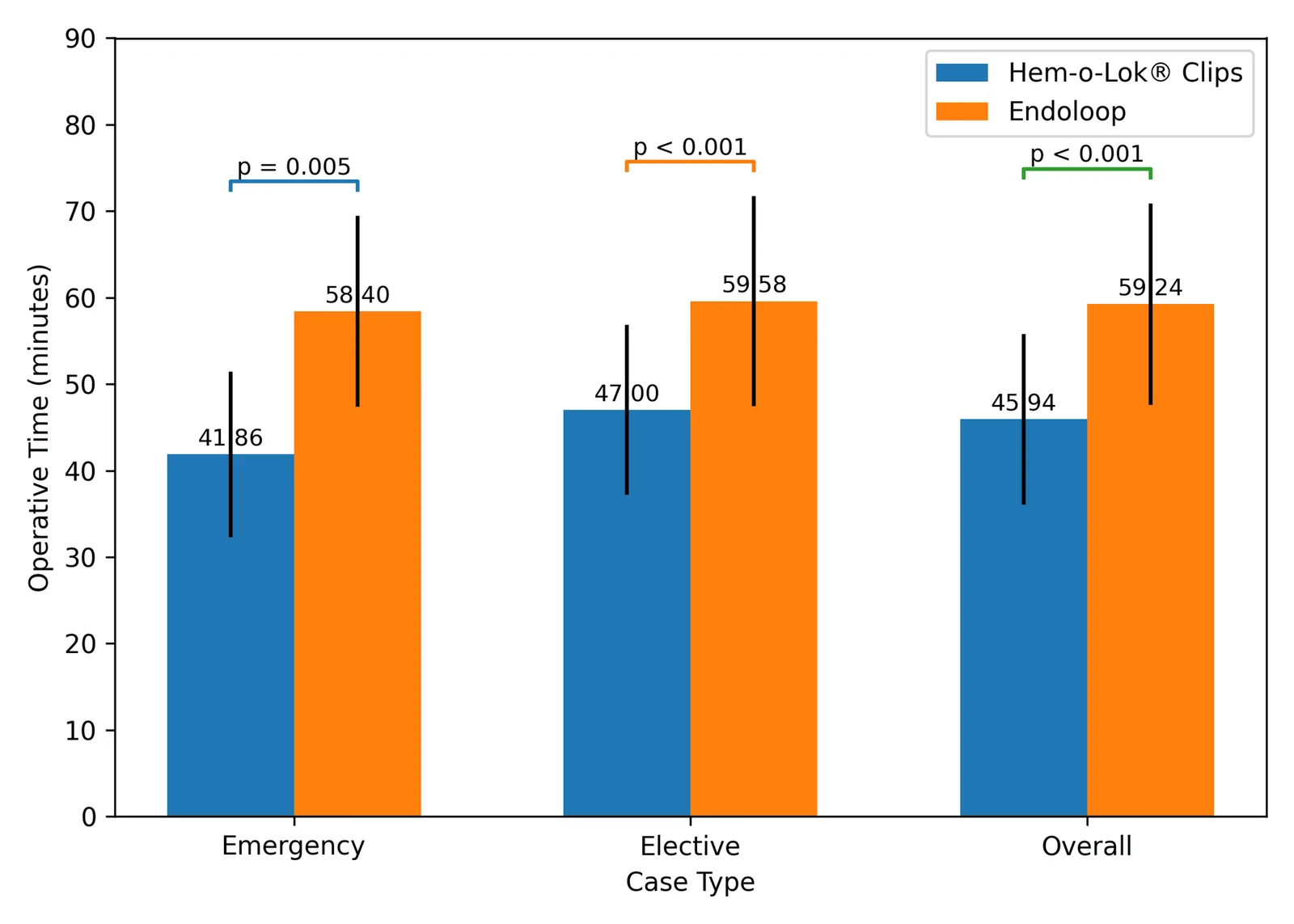
Mean operative time (minutes ± SD) by stump closure technique, stratified by case type. SD, standard deviation

Mean operative time was significantly shorter with Hem-o-Lok® clips overall (45.94 ± 9.87 vs. 59.24 ± 11.66 minutes; *t* = -5.08, *P* < 0.001; 22.4% relative reduction, Hedges’ *g* = −1.216) (Table 2, Figure 4), and this finding was consistent in both emergency (41.86 vs. 58.40 minutes; *t* = -3.28, *P* = 0.005) and elective cases (47.00 vs. 59.58 minutes; *t* = −4.04, *P* < 0.001). The mean appendiceal diameter was comparable between the two groups, with no statistically significant difference observed. Furthermore, no patient in either group experienced difficulty in the application of the Hem-o-Lok® clips or the endoloop due to appendiceal diameter, inflammatory severity, or tissue quality.

**Table 2:** Operative time by stump closure technique and case type. Independent-samples (Welch) t-test; degrees of freedom (df) = 14 (emergency), 44 (elective), and 64 (overall).

Case type	Polymeric clip, minutes (mean ± SD)	Endoloop, minutes (mean ± SD)	Mean diff. (95% CI)	Test statistic	*P*-value
Emergency	41.86 ± 9.60 (*n *= 7)	58.40 ± 11.06 (*n *= 10)	-16.54 (-27.34 to -5.75)	*t* = -3.28	0.005
Elective	47.00 ± 9.84 (*n *= 27)	59.58 ± 12.12 (*n *= 24)	-12.58 (-18.86 to -6.31)	*t* = -4.04	<0.001
Overall	45.94 ± 9.87 (*n *= 34)	59.24 ± 11.66 (*n *= 34)	-13.29 (-18.53 to -8.06)	*t* = -5.08	<0.001

Patients were permitted oral intake after the return of bowel function, which occurred in the majority of cases on postoperative day 01. Most patients' suture removal was routinely performed on postoperative day 11, in accordance with the institutional protocol. As these postoperative care practices were standardized across both study groups, no statistically significant differences were expected. Therefore, a comparative statistical analysis of these parameters was not performed.

Mean hospital stay was shorter with Hem-o-Lok® clips (4.85 ± 0.89 vs. 5.44 ± 1.11 days; *t* = −2.42, *P* = 0.019; 10.8% relative reduction). Hospital stay did not differ by case type within either group (Hem-o-Lok® clips, *P* = 0.615; endoloop, *P* = 0.428).

No complications were observed over 40 days of follow-up.

## Discussion

In this randomized comparison of 68 patients, non-absorbable polymeric locking ligation system (Hem-o-Lok®) clips were associated with a significantly shorter operative time and modestly shorter hospital stay than endoloops, without any increase in complications - findings that are broadly concordant with the existing literature.

Our cohort's age and sex distribution matched well-established epidemiological patterns of appendicitis, peaking in young adults with a near-equal sex split [13,14], and comorbidity clustering (hypertension with diabetes) and prior caesarean-related adhesions were consistent with reported patterns [15,16]. As these variables did not differ between groups, they are unlikely to have confounded the operative-time or stay comparisons.

The shorter operative time with Hem-o-Lok® clips reproduces the direction of effect seen in most comparative studies, even where absolute times vary by center. Colak et al. and Hue et al. both attributed clip efficiency to its lower technical demand relative to endoloop knot tying [10,11]; larger randomized and retrospective series have similarly found endoloop application to be slower than clip or other non-suture methods, without a difference in morbidity [17-20]. The mechanical simplicity of clip placement, compared with the intracorporeal tension adjustment required for a secure endoloop, plausibly explains this time saving, which was greatest in emergency cases, where reduced operative and anesthesia time is most valuable.

The modest reduction in hospital stay with Hem-o-Lok® clips is consistent with reports linking more efficient surgery to faster recovery [21], and our recovery timeline (early oral intake, POD-11 suture removal) matches expected enhanced-recovery and wound-healing benchmarks [22]. However, other series have found no difference in stay between techniques [10,19], and our stratified analysis suggests case type, not closure method alone, was the larger driver - so this finding should be read as supportive rather than primary.

Regarding safety, the complete absence of stump-related complications in both arms matches existing evidence from meta-analytic data: Poon et al. (18 studies, >2,500 patients) and Kumar et al. (13 trials) both found equivalent complication rates between clips and endoloops, with a shorter operative time and, in some analyses, a lower abscess risk with clips [23,24]. Multiple three- and two-arm comparison studies have similarly found no excess complications with polymeric clip relative to endoloop, stapler, or Roeder's knot [25-28]. Experimental histopathology offers partial mechanistic context - non-absorbable clips provoke less chronic inflammatory reaction than absorbable suture, though animal data on adhesion formation have not translated into a clinical signal in this or other trials [29].

Limitations

This trial has several limitations. It was single-center, which may limit generalizability. Neither the surgeon nor the patient could be blinded, given the nature of the intervention. The trial was restricted to uncomplicated appendicitis, so findings do not extend to perforated or necrotizing disease, and 40-day follow-up, while adequate for early stump complications, may miss rare late events such as adhesive obstruction. Additionally, no cost comparison was performed.

## Conclusions

Hem-o-Lok® clips significantly reduced operative time and modestly reduced hospital stay compared with endoloops for stump closure in uncomplicated laparoscopic appendectomy, with no increase in observed early complications over 40 days of follow-up. These findings support Hem-o-Lok® clips as an efficient, safe alternative to endoloops, particularly where operative efficiency matters most. Multicenter trials with concealed randomization and extension to complicated appendicitis would help confirm and extend these findings.
